# Menstrual cups and cash transfer to reduce sexual and reproductive harm and school dropout in adolescent schoolgirls: study protocol of a cluster-randomised controlled trial in western Kenya

**DOI:** 10.1186/s12889-019-7594-3

**Published:** 2019-10-21

**Authors:** Garazi Zulaika, Daniel Kwaro, Elizabeth Nyothach, Duolao Wang, Emily Zielinski-Gutierrez, Linda Mason, Alie Eleveld, Tao Chen, Emily Kerubo, Annemieke van Eijk, Cheryl Pace, David Obor, Jane Juma, Boaz Oyaro, Louis Niessen, Godfrey Bigogo, Isaac Ngere, Carl Henry, Maxwell Majiwa, Clayton O. Onyango, Feiko O. ter Kuile, Penelope A. Phillips-Howard

**Affiliations:** 1 Department of Clinical Sciences, Liverpool School of Tropical Medicine, Pembroke Place, Liverpool, L3 5QA UK; 2 Centre for Global Health Research, Kenya Medical Research Institute (KEMRI), Kisumu, Kenya; 3 Center for Global Health, Division of Global Health HIV and TB, U.S. Centers for Disease Control and Prevention (CDC), Nairobi, Kenya; 4 Safe Water and AIDS Project (SWAP), Kisumu, Kenya; 5 Ministry of Health, Siaya County, Kenya

**Keywords:** Sexual and reproductive health, Adolescence, Equity, HIV, HSV-2, Pregnancy, School dropout, Clinical trial, Menstruation, Kenya, Study protocol

## Abstract

**Background:**

Adolescent girls in sub-Saharan Africa are disproportionally vulnerable to sexual and reproductive health (SRH) harms. In western Kenya, where unprotected transactional sex is common, young females face higher rates of school dropout, often due to pregnancy, and sexually transmitted infections (STIs), including HIV. Staying in school has shown to protect girls against early marriage, teen pregnancy, and HIV infection. This study evaluates the impact of menstrual cups and cash transfer interventions on a composite of deleterious outcomes (HIV, HSV-2, and school dropout) when given to secondary schoolgirls in western Kenya, with the aim to inform evidence-based policy to improve girls’ health, school equity, and life-chances.

**Methods:**

Single site, 4-arm, cluster randomised controlled superiority trial. Secondary schools are the unit of randomisation, with schoolgirls as the unit of measurement. Schools will be randomised into one of four intervention arms using a 1:1:1:1 ratio and block randomisation: (1) menstrual cup arm; (2) cash transfer arm, (3) cups and cash combined intervention arm, or (4) control arm. National and county agreement, and school level consent will be obtained prior to recruitment of schools, with parent consent and girls’ assent obtained for participant enrolment. Participants will be trained on safe use of interventions, with all arms receiving puberty and hygiene education. Annually, the state of latrines, water availability, water treatment, handwashing units and soap in schools will be measured. The primary endpoint is a composite of incident HIV, HSV-2, and all-cause school dropout, after 3 years follow-up. School dropout will be monitored each term via school registers and confirmed through home visits. HIV and HSV-2 incident infections and risk factors will be measured at baseline, mid-line and end-line. Intention to treat analysis will be conducted among all enrolled participants. Focus group discussions will provide contextual information on uptake of interventions. Monitoring for safety will occur throughout.

**Discussion:**

If proved safe and effective, the interventions offer a potential contribution toward girls’ schooling, health, and equity in low- and middle-income countries.

**Trial registration:**

ClinicalTrials.gov NCT03051789, 15th February 2017.

## Background

Young persons aged 10–24 years (yr.) make up a quarter of the world’s population, contributing 1.8 billion persons of whom approximately 90% live in low or middle-income countries (LMIC). Adolescence is a critical time of psychological and biological change, and advocacy has increased to identify interventions that protect young peoples’ lives [1]. These interventions include ways to protect against sexual and reproductive health (SRH) harms, which are disproportionately high among adolescent girls in sub-Saharan Africa (SSA) [2, 3]. Each year an estimated 14 million girls aged 15-19 yr. give birth [2]. Maternal causes kill more girls in this age group than any other cause [2]. Thus, delaying pregnancy to adulthood is important for women’s reproductive health and infant survival, as well as girls economic and social empowerment [4]. In much of east and southern Africa including western Kenya, where unprotected transactional sex is common, young females are highly vulnerable to sexually transmitted infections (STIs), including HIV which may result in mother-to-child transmission [2, 3]. The burden of young female SRH harms is high for individuals, and on their communities and health services, yet sustainable preventive interventions are lacking. Evidence of a positive association between girls’ education, and their health and economic potential, has strengthened international resolve to improve educational opportunities for adolescent girls. While SRH education has not been demonstrated to have a large impact on SRH harms [5], staying in school has shown to protect girls against early marriage, teen pregnancy, and HIV infection, with schoolgirls reporting less frequent sex, and fewer partners with less age disparity [6–8]. Building on the Millennium Development Goals (MDG), which focused on primary school attendance, the post-2015 Sustainable Development Goals encourage investment in secondary, tertiary and vocational education to build human capital, encourage innovation and spur economic growth [9].

Intervention studies using cash transfer (CT) have demonstrated a protective effect on girls SRH (including HIV, HSV-2) and school indicators [8, 10, 11], although results in other studies have been inconclusive [12, 13]. Dropout before secondary school completion is partly explained by girls’ vulnerability once they engage in premarital sex, which is often a precursor to unintended pregnancy or early marriage [7, 14, 15]. Studies have illustrated adolescent girls’ vulnerability to transactional or coercive sex, to obtain necessities such as soap, sanitary products, and underwear [16–19]. Products for menstrual hygiene management (MHM) are one such necessity, and their accessibility remains a pervasive problem in LMIC. A lack of MHM materials, awareness, and facilities, as well as stigma, negatively impact girls’ school-life [20, 21], and can be a driver of girls’ vulnerability to coercive sex. In western Kenya, 10% of 15 yr. old girls self-reported they obtained money through sex to purchase sanitary products [22]. To better understand girls MHM needs in western Kenya, a pilot study in rural primary schools was conducted measuring girls’ menstrual practices, uptake, and safety of a reusable menstrual cup (MS Pilot Study) [23, 24]. The pilot results demonstrated acceptability of the menstrual cup [25], with a lower prevalence of STI and bacterial vaginosis found at 9 and 12 month follow-up among girls using the cup when compared to controls [26], and good clinical safety [16]. Prevalence of school dropout after 12 months was lower but inconclusive due to the small sample [26].

To verify the results of the MS Pilot Study and examine the efficacy, safety, and cost-effectiveness of different school-based interventions in improving girls’ SRH, schooling, and life-chances in rural western Kenya, a randomized controlled trial was designed with a larger population and follow-up duration. The study is designed to inform evidence-based policy to improve girls’ health, school equity and their life-chances which is summarised in this article.

## Methods/Design

### Design overview

This study is a single site, open-label, 4-arm, school-cluster randomised controlled superiority trial taking place in Siaya County, western Kenya. Schools are the unit of randomisation (clusters), with girls as the unit of measurement. Schools will be randomly allocated into 4 arms using a 1:1:1:1 ratio and block randomisation to minimise bias. Enrolment will open in the first school term of 2017 after trial registration and continue until we reach the necessary sample. Girls will be followed-up through graduation and into employment or up to 10 academic terms to determine if they complete secondary school (Form 4), see Additional file 1: Spirit Checklist.

### Primary objective

To determine the impact of menstrual cups or CT alone, or in combination, on a composite of deleterious outcomes (HIV, HSV-2, and school dropout) when given to secondary schoolgirls in western Kenya.

### Secondary objectives

1. To measure the age-specific differences in the acquisition of HIV and HSV-2 infections in secondary schoolgirls and risk factors for incident HIV and HSV-2 infections.
2. To determine the risk, risk factors and reasons for dropout and other school indicators among secondary schoolgirls examining the influence of social, epidemiological, and/or health characteristics.
3. To determine the cost benefit of menstrual cup and CT programmes for schoolgirls by assessing the cost savings of outcomes averted, for individual and combined interventions, and resulting societal impact.
4. To determine the safety of menstrual cup use, including risk of cup contamination over time, serious adverse events, and identify factors that increase or modify this risk.
5. To determine factors affecting how adolescent girls spend CT money, and what training is required to support their financial literacy and decision-making.
6. To determine any adverse outcomes associated with CT and evaluate ways to mitigate risk.
7. To determine the impact of the interventions on girls’ sexual behaviours, including age of sexual début, coerced sex, number of partners, age of partners, pregnancy, condom use and use of contraception.
8. To examine programme implementation for interventions in schools, working with beneficiaries and stakeholders to develop programme implementation packages.

### Design considerations

#### Why secondary schoolgirls?

Among schools located in our proposed study area, the dropout rates are higher among girls in secondary when compared to primary school girls. Unpublished school enrolment data for 2015 in the study location shows that only 26% of primary school girls drop out of primary school compared to 36% who drop out of secondary school (local school enrolment data, unpublished). These high dropout rates for secondary schoolgirls exert a high burden on the national economy; it is estimated that girls would contribute 48% more of annual GDP to their economies over their lifetime [4]. Our pilot study found that following the abolition of primary school fees 6 years ago, girls complete primary at a younger age (< 15 yr). Thus, fewer girls in primary school reach menarche and sexual debut. While prevalence of HIV was low in our primary school cohort (< 1%), health studies in the same study area have documented very high HIV incidence for secondary school-aged girls, who range in age from 13 to 30. In one study, HIV prevalence was 8.8% in 15-19 yr. olds, sharply rising from 1.3% among 13-14 yr. olds to 3.3% in 16 yr. olds and 12.8% in 18 yr. olds [27]. A similar steep increase by age was seen in HSV-2 prevalence [27]. A high prevalence of STIs was detected in 15-19 yr. old girls in neighbouring Kisumu [28]. In pilot study focus group discussions (FGDs), when asked about reasons for drop out, girls voiced reasons linked to exposure to sexual activity (resulting from alcohol, funeral parties, needs for money, and coercive sex), and these were more frequently stated among older girls [17]. During these FGDs, girls were able to vocalise their concerns about pregnancy risks, and issues around lack of money for school and personal needs. They reported that their menstrual needs were unmet but a high priority and at times compelled them to have sex to obtain money to buy pads [17]. In a separate study in the same area, 10% of 15 yr. olds surveyed reported they had sex for money in order to purchase sanitary pads [22].

#### Justification for a composite endpoint

The primary composite endpoint will include incident HIV, HSV-2, and school dropout in girls sero-negative or for both HIV and HSV-2 on enrolment or undetermined sero-status at enrolment (conservatively the sample size allows for 20% refusal for testing at baseline). The presence of HIV or HSV-2 at enrolment precludes the components from contributing to the primary composite endpoint. Thus, among HIV-negative girls who were HSV-2 positive on enrolment only incident HIV infection and school dropout would contribute to the primary endpoint; among girls who are both HIV and HSV-2 positive on enrolment, only school dropout would contribute. This endpoint represents the key drivers compromising girls’ health and life chances into adulthood. The rationale behind this composite endpoint is to increase the power for a given sample and to build a single outcome across all girls regardless of their independent HIV and/or HSV-2 status at enrolment.

#### Justification for cumulative school dropout

The cumulative risk of school dropout among secondary schoolgirls is an acute problem in western Kenya; 36% of girls drop out before the start of the fourth and final year of secondary school due to teen-age pregnancy, lack of school funds, illness, work or family commitments, or viewing school as unnecessary [17]. The need for MHM is also perceived as a constant stressor impacting school-life given that traditional MHM items (rags, paper, etc) leak, cause odour and discomfort, and cause girls to habitually miss school and fall behind. Some evidence suggests that poor MHM even leads some to engage in transactional sex for essential ‘luxuries’ such as pads and soap [17, 22]. During the pilot study [23–25], we found that use of MHM products (reusable menstrual cups or pads) for at least 1 year had the potential to prevent school dropout [26]. CT programs also have shown potential for CT improving the odds of being enrolled in and attending school, improving household socio-economic status (SES) and quality of life, and reducing early marriage [29, 30]. In the trial, we define dropout as not attending school consecutively for at least 1 term or longer. Girls who attended part or all of Form (class) 4, but then do not sit the final national Kenya Secondary Certificate of Education (KSCE) exams will be considered a dropout in that final term. Other secondary school indicators such as grade repetition will also be documented. Girls who return to school after being classified as a dropout will be classified as re-enrolled.

#### Justification for cumulative risk of incident HIV and HSV-2

Risky sexual exposure can cause harm to a girl’s sexual and reproductive health and negatively affect her life-chances even while remaining in school. The pilot showed high prevalence of laboratory confirmed STIs in this rural area in western Kenya even among primary schoolgirls (28% of girls had reproductive tract infections, predominantly bacterial vaginosis) [26, 31]. Community surveys in the pilot site found an HIV prevalence of 11% among females under 30 yr., rising from 1% in 15 yr. to 20% by age 29 [22, 27], and a reported 52% of girls in this area engaging in transactional sex for money, gifts or services [32]. HSV-2 is the most common cause of genital ulcer disease worldwide, the most prevalent STI in sub-Saharan Africa, and a well-established biomarker for sexual risk behaviour [8, 33, 34]. Evidence suggests HSV-2 prevalence in girls in the study area increases from 10% in 13-14 yr. to 28% in 15-19 yr., and 70% among the 20-24 yr. [27, 35].

A trial in Malawi that provided CT to school girls aged 13-22 yr. found that HIV and HSV-2 prevalence were 33 and 70% lower respectively in CT recipients after 18 m intervention when compared to controls [8]. Results were supported with reduced frequencies of self-reported sexual activity and less age discordant sex [8]. The impact of menstrual cups on HIV or HSV-2 has not been evaluated, but when assessed during the pilot study, cups were associated with a lower prevalence of STIs and bacterial vaginosis [26], both important risk factors for HIV acquisition and transmission [36–38]. This information corroborates reported narratives that control-arm girls most acutely felt the need to have sex to obtain sanitary pads [24, 25]. Laboratory confirmation of infections is essential, however, as girls and young women’s reported symptoms are poorly predictive of infection [31, 39–43].

### Study setting

**Fig. 1:**
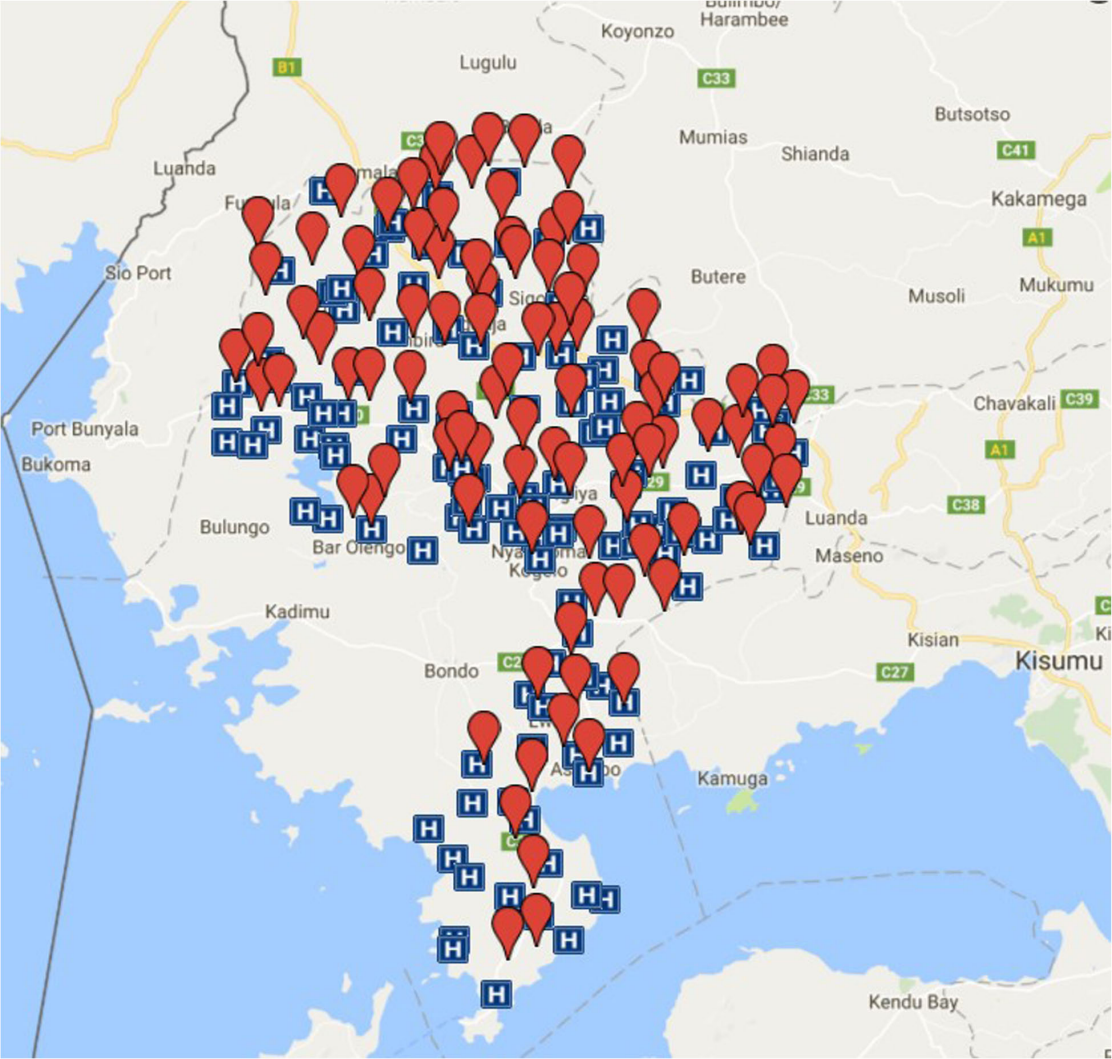
Map of Siaya County Public Health Facilities and 96 CCG Study Schools

The study will be conducted in schools in Siaya County in rural western Kenya, extending to contiguous areas that include Kisumu County if needed. The site is in a health and demographic surveillance system (HDSS) positioned 400 km west of Nairobi, with its southernmost point reaching Lake Victoria [44]. The population are mostly members of the Luo ethnic group, and are mainly subsistence farmers [45]. Siaya is an impoverished area, with previous studies estimating households have a mean annual income approximating $600 to $700 [46]. An estimated four out of ten child learners miss school daily in Siaya County [47]. Gender equity seen in primary school falls during adolescence, with between 25 and 33% more boys than girls attending secondary school by age 18 [48, 49]. The disease burden typifies rural African communities [44], with mortality in adolescents and young adults attributed to communicable diseases, injuries [50], and maternal causes [51]. Advances in antiretroviral therapy (ART) access have been associated with reducing adolescent and young female mortality by half [50, 52]. Physical and sexual violence against females is one of the highest in Kenya, with 12% of women reporting their first sexual intercourse was coerced [53], rising up to 45% among adolescent girls [54]. The former pilot study evaluating menstrual interventions was conducted in one of the three sub-divisions within the study area, with water, sanitation, and hygiene (WASH) observations illustrating presence of latrines and water, but not soap in schools [23]. The menstrual care among the population was examined and illustrate girls’ and young women’s preference for commercial pads over traditional items; with 10% of 15 yr. olds reporting they had sex for money to purchase sanitary pads [22]. Schools and health facilities have been geo-located (see Fig. 1, below).

### Eligibility criteria: schools

#### Inclusion criteria for schools

- Secondary school within study area
- Girls or co-educational school
- Day school
- Approval by Head Teacher

#### Exclusion criteria for schools

- Boys only school
- Boarding schools
- Special needs schools (i.e. for the blind)

### Eligibility criteria: participants

#### Inclusion criteria for participants

- Attend secondary day schools in the study area
- Resident of the study area
- Have a history of established menses (> = 3 times)
- Have no disability preventing participation
- Assent to participating in the study and have received parent or guardian consent

#### Exclusion criteria for participants

- Attend boarding schools
- Visibly pregnant or declare pregnancy at baseline (girls who don’t declare pregnancy but whose delivery dates confirm pregnancy started prior to enrolment will be excluded from the analysis.)

### Trial interventions

Schools will be randomised to one of 4 arms:

1. One menstrual cup with training on safe use and care, with handwash soap termly.
2. Cash transfer (CT) Ksh 1500/term plus financial literacy training.
3. Combined menstrual cup and CT with training on financial literacy and cup safe use and care.
4. ‘Usual practice’ control (control arm), with handwash soap termly.

All participants regardless of school cluster will receive puberty and hygiene education.

#### Menstrual cup

The menstrual cup is a medical grade silicone bell shaped container which is inserted into the vagina to collect menstrual flow, and requires emptying at regular intervals (4–8 h) [55]. Cleaning by boiling is recommended at the end of each cycle. The Mooncup® will be used in the trial [56], selected because it has been tested in the UK [57, 58] and internationally [26, 59], is produced to ISO 13485:2003 standards, and registered by the U.S. Food and Drug Agency of Medicines (FDA; Registration Number 3009117944); and was successfully used in the pilot study [54]. Further, its’ white colour when new, changing to brown after use, allows physical observation of use [26]. Girls will receive school-based training on safe cup use and care (including insertion, emptying, re-insertion, cleansing, and storage). The trial will document girls’ use over time.

#### Cash transfer pocket money

Cash transfer (CT) programmes are a popular social protection tool in developing countries that aim, among other things, to improve education outcomes and reduce risky sexual behaviour [8, 10, 11, 60, 61]. A sum of US$5 per month (~Ksh500; exchange December 2015) was recommended for future studies. CT programmes which were conditional on attendance have been shown to improve school outcomes more than unconditional or non-monitored [29]. This trial will provide Ksh1500 (US$15) per term (3 terms per school year) for up to 10 academic terms. Conditionality for CT receipt will be based on 80% or more school attendance in the previous term, in line with other studies [8, 61–64]. After assent, participants in the CT arms will receive school-based financial literacy training and a bank card. For this trial, Equity Bank pre-paid cash cards will be used for minors after obtaining guardian consent. Girls must provide a birth certificate and a guardian ID to receive a bank card. Precautions will be taken to ensure girls have direct access to their accounts but maintain low visibility to minimize the risk of theft, harassment, or violence. School registries will be assessed retrospectively per term to verify school attendance, with spot-checks conducted to minimise risk of falsification of registries. The trial will document girls’ use and spending choices over time.

### Endpoints / outcome measures

Primary outcome:

Composite: incident HIV, HSV-2, all-cause school dropout by the end of the study.

Secondary outcomes:

- School dropout
- HSV-2 incidence
- HIV incidence
- Reported sexual behaviour indicators (including age at sexual debut, age-discordance of partners, coercive sex, number of sexual partners, pregnancy, condom use, and use of modern contraceptives)
- School performance indicators (Kenya Certificate of Secondary Education [KCSE] results, grade repetition, prevalence of re-enrolment, and absenteeism)
- Quality of life using EuroQoL and PEDSQL
- Cost-effectiveness of interventions from the societal, including girls’, perspective

#### Safety endpoints

- Tolerability: any adverse events assessed in a general health questionnaire
- Primary Safety:
  - Toxic Shock Syndrome
  - Violence associated with interventions provided
- Secondary Safety:
  - *E. coli* growth on sampled cups
  - Other emergent harms that may occur with provision of cash pocket money or cups.

### Sample size estimates

Original trial design sample size estimate: Sample size and power calculations were performed for the minimum number of schoolgirls needed in the proposed 4-arm trial using sample size calculation software (NCSS/PASS); calculations were validated using SAS based simulation studies. Five primary comparisons of the primary endpoint were tested: [1] menstrual cup vs usual practice, [2] CT vs usual practice, [3] combined CT and cup vs usual practice, [4] combined CT and cup vs menstrual cup only, and [5] combined CT and cup vs CT only. Calculations were based on a 2-sided alpha of 0.01 to allow 5 primary comparisons of interest, assuming an ICC value of 0.008. Taking a target of mid-late Form-1 of schools in the study area gives a sample size average of 46 girls, a 1 yr. enrolment period, a 5% overall refusal to take part in the study, 20% refusal at enrolment to consent to HIV testing among participating girls, an average of 10 terms (~ 3.3 yr) follow-up through the end of Form-4, and 20% loss to follow-up or refusal to provide biological samples at the end of the study period. Of 46 enrolled girls/school, on average, 35 (0.95*0.80 × 46) will contribute to the primary analysis; we assume that 6.9 will be HSV-2 or HIV positive on enrolment (24.7% of 28 girls who agree to testing) and the remaining 28.1 will be HSV-2/HIV negative (*n* = 21.1) or of unknown HSV-2/HIV sero-status (*n* = 7) because no assent/consent was provided for testing at enrolment. With these assumptions, a trial with 4 arms of 21 schools per arm (84 schools total) enrolling 46 girls/school (i.e. 966/arm; 3864 girls total) with 35 girls/school contributing to analysis, will have 90% power to detect a 25% reduction (Relative Risk [RR] = 0.75) in the 3.3 yr. incidence risk of the primary endpoint from 44.1% in the control group to 33.1% with either intervention, and 80% power to detect a 22.2% reduction (RR = 0.778) to 34.3% (both at alpha = 0.01).

*Source data:* The ICC value of 0.008 was the observed ICC value for the composite endpoint of school dropout and STIs in our previous pilot study, and 0.0084 for school dropout alone [26]. The anticipated effect sizes of 25% (RR = 0.75) for the primary endpoint is based on a model combining the impact and event frequency of the 3 components of the primary endpoint in the three strata: HSV2/HIV negative girls (60.2% of the overall sample), HSV-2 or HIV positive girls (19.8% of the sample), and girls for whom the sero-status is unknown (20% of the sample). The model predicts that a 25% (RR = 0.75) overall reduction from 44.1 to 33.1% with single interventions, or from 33.1 to 24.8% with the dual intervention can be achieved with the following combination of relative risk reductions for school dropout and HIV and HSV-2 incidence respectively: 30 and 25.7%; 25 and 34.2%; or 20 and 43.0%. The anticipated minimum reduction of 30% in dropout is based on an average 31% reduction in a meta-analysis comparing controls against cash transfer [29], and is more conservative than a 58% reduction observed with menstrual cups in year-2 of the pilot [26]. The 48.8% reduction in HIV and HSV-2 incidence is based on a 51.9% reduction observed in year-2 of the pilot study, adjusted for the fact that baseline HSV2-HIV status would not be available for 12 of 60 girls/school (20%) on enrolment, 3 to 4 of whom may have undetected HSV-2 or HIV on enrolment. The observed relative risk reduction in the HIV and HSV-2 incidence was 48.8% (based on a 43.4% reduction in STI prevalence by the end of the previous pilot in 2014) [26].

### Blinded sample size re-estimation

A blinded sample size re-estimation was conducted in 2017 using the baseline data from all arms pooled to validate the assumptions made during the original sample size estimations in the trial design phase. The average number of girls per school and the baseline prevalence of HSV-2/HIV (a proxy for the anticipated incidence) were lower than anticipated. A sample size re-estimation with pooled data demonstrated that a total of 96 school clusters (24/arm) are required with an anticipated average of 41.25 girls per cluster to obtain 90% power to detect a 25% (RR = 0.75) reduction in the primary endpoint from 39.3% in the control arm to 29.5% in any of the 3 intervention arms (alpha = 0.0167 allowing for 3 primary comparisons against the control arm), with an ICC of 0.008 and allowing for 20% loss to follow-up. This yields a full sample of 3960 overall, 3168 of whom are expected to contribute to the primary endpoint, 33 per cluster. This same sample size provide 80% power to detect a 25.7% (RR = 0.743) reduction from 29.5% in any of the single intervention arms to 21.9% in the combined intervention arm (alpha = 0.025). The total sample size may exceed 3960 if the average cluster enrolled has more eligible girls than anticipated as the intention is to give every eligible girl in each secondary school the opportunity to participate.

### Assignment of interventions

#### Allocation

School clusters are the unit of randomisation and girls the unit of measurement. A census of secondary schools in the area will be used to select the eligible schools. The trial statistician will produce block randomized groupings of four schools (blocks) using a 1:1:1:1 ratio and based on location and size, including larger schools (e.g. with more than 20 target girls) for logistical reasons. Arm allocation of schools to intervention arms will be achieved using community ceremonies. During a public ceremony, head teachers representing their respective school will be called up with the rest of their blocks for balanced randomization. The head teachers will each simultaneously pick 1 of 4 coded items, and once all blocks have completed this process and all schools have been randomly allocated, study arm will be displayed by opening sealed envelopes and breaking the code. This methodology was informed by the pilot study where randomisation ceremonies with head teachers were successful [26].

#### Blinding

Participants cannot be masked to their treatment arm due to the nature of interventions provided. Laboratory personnel testing for HIV and HSV-2, investigators, and trial statisticians will be blinded to the study arm. Field staff will be masked as much as feasible, including those who conduct home visits to confirm dropout. Bias will also be minimised by use of block randomisation stratified by school size. An independent person will prepare the sealed envelopes with the study arm allocation. Study arm allocation will not be recorded in the central database to ensure the trial statistician and data managers remain blinded throughout the study. This information will be recorded separately and only be merged with the main database following approval of the statistical analysis plan (SAP), closure of the databases and submission of a copy to the independent statistician of the Data Monitoring and Ethics Committee (DMEC).

### Participants’ timeline

#### Overview

**Fig. 2:**
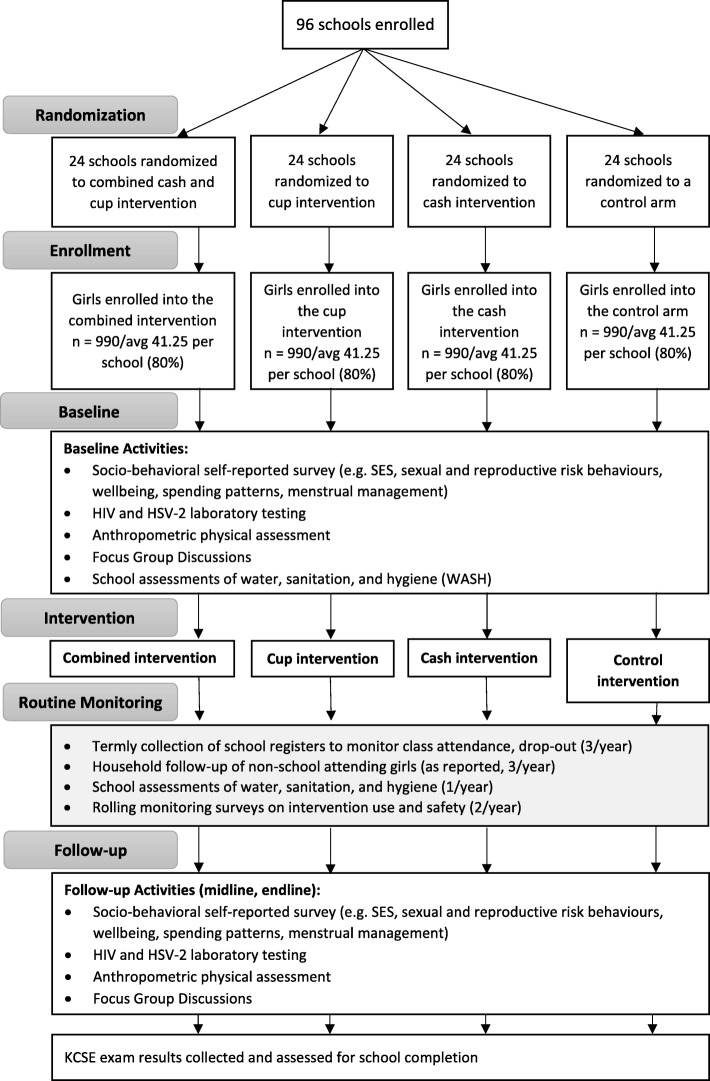
Flowchart of Randomization and Study Design

The participant’s timeline will commence after pre-recruitment preparations, including ministry, school, and community stakeholder meetings and approvals. Parent consent for their daughters’ participation will follow the school cluster randomisation ceremonies. Participants’ meetings for assent, pre-screen enrolment and baseline screening, mid-line screening (second study year), and end-line screening (third study year) will be held as ‘Health Days’ in randomised schools (Fig. 2).

#### Pre-screen enrolment, assent and baseline screening

The school enrolment register will be used as the sampling frame to define all target girls in the study schools. Parents of all girls will be approached at the end of the school information meeting to request informed consent for [1] the main study, [2] HIV testing and counselling, and [3] blood storage. Signing will be private and one-to-one with a trained member of the study team. The enrolment list will be updated at the meeting to record girls transferring out or into the school and missed parents will be followed up at home for consent. Reasons for non-consent will be documented.

Girls whose parents have consented to their participation in the study will be informed of the study purpose and procedures. Each girl will individually be asked to give her assent to participate and asked key eligibility criteria questions (see eligibility criteria: participants, above). Girls who meet the eligibility criteria will then be invited to participate in the study.

Participants will privately self-administer a combined demographic, social, behavioural and quality of life/wellbeing questionnaire using tablets during the ‘Health Day’; absent girls will be invited to participate at a subsequent ‘Health Day’ when logistically feasible. All relevant information will be captured in the survey questionnaire. Baseline questions around demographics, use of menstrual items, and access to cash and personal bank account will be asked, as well as other secondary outcomes. Wellbeing will be assessed using the adolescent (12-18 yr) 23-item PedsQL™ 4.0 (Paediatric Quality of Life Inventory; http://www.pedsql.org/), and will measure physical, emotional, social, and school functioning of children, core dimensions as delineated by WHO [65].

A baseline clinical survey will be conducted to define pre-intervention HIV and HSV-2 prevalence, and height, weight, and waist measures of participants. Documentation of population HIV prevalence is important to understand frequency of mother-to-child transmission of HIV, noted in a Zimbabwean schoolgirl CT study which only evaluated HIV and HSV-2 at endline [30]. However, refusal to have an HIV or HSV-2 test at baseline will not preclude participants from joining the study. School ‘Health Days’ will be operated with a trained mobile team at a location at or close to their school. All sample collection and HIV counselling will be conducted by a team of trained HIV Testing and Counselling (HTC) staff. Results will not be given then, but separately to participants on an individual level at the health clinics with trained counsellors and testing and counselling and care facilities. Participants can visit clinics individually without peer pressure and are encouraged but not obligated to ask their parents to accompany them. If consent/assent has been obtained for blood collection, girls will provide 600uL of blood for HIV and 1.5 ml for HSV-2 with any blood not used for these two tests stored as dried blood spots for future testing of other STIs or vaccine preventable infections, if funding allows. Blood will be collected through fingerpick and stored and transported in Microtainer EDTA tubes to KEMRI laboratories for analysis. Blood will be stored for a maximum of 5 yr., after completion of the trial, after which it will be destroyed.

#### Midline screening

All participants will be invited to participate in a mid-study behavioural survey to update socio-behavioural characteristics, including marriage status, sexual exposures, and document patterns of intervention use, problems encountered, and any possible safety issues. Midline HIV/HSV-2 testing will be conducted mid-study. These tests and follow-up counselling and treatment will follow the same methodologies used at baseline. These measurements will allow closer examination of incidence over time and offer the opportunity to test and counsel participants who exit the study before the endline survey. Baseline consents and assents include this assessment. Participants are reminded they have the freedom to withdraw or refuse testing.

#### End study screening

Similar to the baseline Health Day, participants will attend an end of study Health Day to complete an endline behavioural survey to document changes in socio-behavioural characteristics (including risky sexual behaviours, quality of life/wellbeing measures) and intervention use, problems encountered, and any perceived harms. Outreach activities to survey enrolled girls who have left school or dropped out will be conducted if funding is available. HIV and HSV-2 serostatus will be assessed at this same Health Day to determine incidence among those testing negative at enrolment and during interim follow-up testing. Careful consideration and coordination with head teachers will be needed to secure the dates for the final survey to ensure no disruption for girls in Form 4 while they take final exams. Endline HIV/HSV-2 testing will be conducted on Health Days in safe spaces among all enrolled girls to protect the confidentiality of baseline HIV positive participants. Participants are reminded they have the freedom to withdraw or refuse testing.

#### Unscheduled visits

School dropout will be assessed every term until the study end. Regular monitoring of school registers will be conducted to determine dropout among participating girls. Girls who dropout will be followed-up with an unscheduled visit to the home to understand reasons for dropout and confirmation of the same, and to identify those that have migrated to a different area who may still attend school (e.g. are classified as loss to follow-up). An unannounced annual WASH survey will be conducted at all participating schools, to observe the presence and state of latrines, water availability, water treatment, handwashing units and soap. At any time, participants displaying adverse events will be assessed by a study nurse, with a triage form evaluating seriousness and potential relationship with the interventions and tailored AE and SAE forms to document relevant details (see Additional file 2: SAE Report Form).

#### Focus group discussions

FGDs will be held pre-intervention, annually (interim) during the trial, and at the end of the study. Feedback from these will document participant and other beneficiaries understanding of the interventions, use, impact and any problems arising.

### Laboratory procedures

#### Clinical testing

HIV testing will be conducted in accordance to Kenyan national guidelines [66]. HSV-2 will be examined using Kalon gG2 ELISA test kits (Kalon Biologicals Ltd., Guilford, UK), with quality assurance performed. Any additional blood collected at baseline, interim, or end of study will be stored for future testing of other STIs or vaccine preventable infections, if funding allows.

#### Cup contamination

A register of all participants receiving cups will be used to randomly select a swatch of used cups by duration of provision. This will exclude girls who received a replacement cup due to loss, theft, or damage. Randomly selected participants will be traced and asked if they are willing to swap their existing cup for a new one, to allow laboratory examination of their cup. Each used cup will be placed in a separate lock- bag labelled with participant ID and transported to the laboratory and tested for *E coli* growth. Cups will be swabbed using polyester tipped swabs moistened in normal saline and inoculated into both MacConkey (MAC) agar and blood agar (BA) and incubated for 18–24 h at 37 °C. After incubation, colony types will be visualized for characteristic morphology of *E.coli* and others from the MAC plates, and subjected first to analytical profile index (API) testing for suspected *E coli* growth [67], then incubated for 18–24 h at 37 °C*.* The results will be interpreted using API software [68].

### Statistical methods

A study statistical analysis plan will be developed during the course of the study for the final analysis. This will be completed prior to the unblinding of data at database lock.

#### Screening failures

A participant who gives informed assent (after parental consent) and is provided with a study ID, but then is found not to fulfil the eligibility criteria, will be classified as a screening failure and excluded from the intention-to-treat (ITT) and the per protocol (PP) analysis. Pregnant girls who do not declare pregnancy at enrolment will be excluded from analysis after the dates of normal (or otherwise) deliveries confirm that the current pregnancy was ongoing at enrolment.

Intention-to-treat (ITT) population.

The intention-to-treat population (the full analysis population) is defined as all participants who provided parental consent, themselves assented, and were enrolled into the study. These girls will be included in the intention-to-treat analysis regardless of whether they have completed all endline evaluations.

Per protocol (PP) population.

The per-protocol population within the menstrual cup groups is defined as all participants receiving the cup with evidence showing actual use. For cash transfer, ‘per protocol’ constitutes all girls receiving the cash intervention until dropout or reaching the endpoint. Participants documented to have crossed over between school clusters will be excluded.

#### Cost-effectiveness analyses

An economic evaluation will be conducted to provide evidence for the cost-effectiveness of the three interventions. This will be used to estimate the societal cost consequences and efficiencies of the intervention packages to inform health service delivery and future policy decisions.

#### Safety outcomes

Adverse events (AEs) and serious adverse events (SAEs) will be monitored, managed and recorded during the study (see Additional file 2: SAE Report Form). AEs will be reported and tabulated for each treatment arm, overall, and according to body system on a per protocol basis. Intervention emergent AEs are defined as adverse events that had an onset on the day of the intervention, or thereafter. AEs that have missing onset dates will be considered to be treatment emergent. No formal statistical testing will be undertaken. All laboratory data will be listed and summarised.

### Ethics approval and consent to participate

This protocol, the informed parent consent and participant assent documents, and participant information sheets have been reviewed and approved by the Research Ethics Committees at the Kenya Medical Research Institute, Nairobi, Kenya (KEMRI protocol #3215) and the Liverpool School of Tropical Medicine, Liverpool (LSTM protocol #15–005). The Centers for Disease Control and Prevention gave approval for reliance on the KEMRI IRB (2016–136). Registry approval for trailing menstrual cups was given by the Kenyan Poisons and Pharmacy Board (ECT_16_07_06). Annual renewal of approvals by KEMRI, LSTM, and KPPB are required based on reporting of trial activities in the prior year.

## Discussion

In this study we are hypothesizing that, as a result of receiving the trial interventions, participating adolescent girls’ health and schooling will improve. Prior studies have illustrated that the provision of a menstrual cup can lower rates of reproductive tract and sexually transmitted infections [26]; and that the provision of cash transfer impacts positively on girls’ schooling outcomes [8, 13]. Moreover, evidence is building that enrolment and consistent attendance in school acts as a social vaccine with multiple benefits for girls [6, 15]. This trial will determine if provision of a menstrual solution alone, or in combination with cash transfer directly to schoolgirls can improve their life chances, in terms of reducing their risk of HIV, STI (HSV-2), and school dropout. In our trial, we postulate the interventions tested (cups alone, CT alone, or cups and CT) will lead to a reduction in schoolgirls’ exposure to sexual and reproductive harms, while increasing their opportunity to complete their schooling, compared with controls. Enrolment, intervention and follow-up of participants across a wide geographical area in rural Africa requires a strong collaboration with schools, communities and organisations. The collaboration in western Kenya between KEMRI, LSTM, SWAP, CDC and government of Kenya (GoK) provides this. Parallel small group sessions evaluating programme fidelity and uptake will inform and strengthen the development of programmatic materials for implementation, should the trial show positive outcomes.

Our research will be communicated to the UK and Kenyan public, Kenyan local, county and national ministries, NGO and aid agencies, national and international universities, research groups, international development and aid agencies, donor organizations, and international agencies setting global policy. We will use multiple communication strategies to target information to the correct audience, as appropriate. Much will be through face-to-face interactions at workshops, meetings, local forum presentations, and international conferences. Communication through technology transfer will be used to disseminate more widely to a broader audience, through online networks, webinars, online news, blogs, and publication portals.

We hope that if the interventions prove to be successful and our communication strategy sound, this trial could contribute to improved retention of adolescent girls in school, and could have multiple benefits for health and education services, and national and global level development. This growing evidence base must be used to help girls complete their educations and become financially independent adults, better manage their own menstrual hygiene, and reduce the negative psycho-social pressures and stigma leading to sexual exploitation, violence, illness, premature marriage, and death during childbirth. Cascading benefits may include that communities will benefit from an increase in social capital, and a reduction in resources required to support unemployed, sick, and pregnant girls. Evidence-based-policy will lead to schools being beneficiaries, by improving girls’ experience of menstrual care in school; and teachers will benefit from girls’ improved attention in class and equitable teaching. As more girls complete education, there will be greater opportunity for training female teachers, redressing the gender imbalance. More engaged pupils will increase teachers’ job satisfaction and better grades will raise school profiles. Partnerships between education and the health sector will be strengthened. Economic benefits would translate nationally; for example, researchers estimate that in Kenya, if all 1.6 m adolescent girls were able to complete secondary school, and the ~ 220,000 girls who were pregnant and delivered could be educated, there would be a cumulative effect adding up to £2.1 billion towards Kenya’s gross income per year [4]. Implementation of successful interventions globally will increase the number of girls completing school, reducing the current global estimate of 44 m adolescent girls out of school. Implementing interventions that retain girls through secondary school will have global economic benefits, as it is estimated that countries growth rates would increase on average by ~ 1% annually if girls’ education was raised one level higher (i.e. secondary status). Interventions will reduce the prevalence of teen births and poor maternal outcomes, and the rate of new HIV infections in adolescence which currently account for ~ 40% of new infections. This will decrease the burden of HIV programme costs for antiretroviral drugs and antenatal care to prevent mother to child transmission.

## Supplementary information

**Additional file 1.** SPIRIT Checklist

**Additional file 2.**SAE Report Form

## Data Availability

Not applicable: Out manuscript does not contain any data or related findings.

## Abbreviations

95% CI: 95% Confidence Interval
AE: Adverse event
AIDS: Acquired Immunodeficiency Syndrome
ART: Antiretroviral therapy
CDC: Centers for Disease Control and Prevention
CHW: Community Health Worker
CRF: Case Record Form
CRO: Contract Research Organization
CT: Cash transfer
DfID: Department for International Development, UK
DHSC: UK Department of Health and Social Care
DMEC: Data Monitoring and Ethics Committee
DSMB: Data Safety Monitoring Board
ELISA: Enzyme linked immunosorbent assay
ERC: Ethics Research Committee
FDA: Food and Drug Administration
FGD: Focus group discussions
GCP: Good Clinical Practice
GEE: Generalised Estimating Equation
GMP: Good Manufacturer Practice
HDSS: Health and Demographic Surveillance System
HIV: Human immunodeficiency virus
HSV-2: Human simplex virus type 2
HTC: HIV testing and counselling
IDI: In-depth interviews
IRB: Institutional Review Board
ITT: Intention to Treat
JGHT: Joint Global Health Trials
KEMRI: Kenya Medical Research Institute
LSTM: Liverpool School of Tropical Medicine
MHM: Menstrual hygiene management
MoEST: Ministry of Education, Science and Technology
MoH: Ministry of Health
MRC: Medical Research Council, UK
PCR: Polymerase Chain Reaction
PE: Protective efficacy
PP: Per protocol
QoL: Quality of life
RCT: Randomised Controlled Trial
REC: Research Ethics Committee
RR: Relative risk
RTI: Reproductive tract infections
SAE: Serious adverse event
SAP: Statistical Analysis Plan
SOP: Standard Operating Procedure
SRH: Sexual and reproductive health
STI: Sexually transmitted infections
SWAP: Safe Water and AIDS Project
tCTU: Tropical Clinical Trials Unit
TSC: Trial Steering Committee
WASH: Water, sanitation and hygiene
WHO: World Health Organization
