# Supplementary material for: Menstrual cups and cash transfer to reduce sexual and reproductive harm and school dropout in adolescent schoolgirls: study protocol of a cluster-randomised controlled trial in western Kenya
Source: BMC Public Health. 2019 Oct 21;19:1317. doi: 10.1186/s12889-019-7594-3 (PMC6805471; doi:10.1186/s12889-019-7594-3)
Supplement: Supplementary file 2 — Additional file 2.SAE Report Form [file 12889_2019_7594_MOESM2_ESM.pdf]

| Cups or Cash for Girls (CCg) Trial | | ***Serious Adverse Event Form*** Sponsor: Liverpool School of Tropical Medicine, lstmgov@lstmed.ac.uk, pv@lstmed.ac.uk | | | |
|---|---|---|---|---|---|
| **Participant ID** | | **Date of report** | __ __ - __ __ __ - __ __ __ __ | **SAE no.** | |

| | | | |
|---|---|---|---|
| **Type of report** | ☐ **Expedited** | ☐ **Full** | |
| **SECTION A: PATIENT DETAILS** | | | |
| **Age (years)** ☐ | **Or date of birth (DD-MMM-YYYY)** | __ __ - __ __ __ - __ __ __ __ | |
| **Relevant medical history including pre-existing medical conditions** | | | |
| **Informant relationship to participant i.e mother; clinic nurse; teacher; etc** | | **Contact details of informant (phone number, address etc)** | |
| **SECTION B: SERIOUS ADVERSE EVENT AND INVESTIGATIONS** | | | |
| **Main diagnosis** | | | |
| **Description of event (include details of all signs, symptoms and events that have occurred at the time of the main event)** | *In cases of violence, please describe the nature of the violence and the clinical outcome for the patient i.e. any bruises, broken bones, cuts etc. Indicate on the diagram in appendix A the type and location of injuries on the body provided.*<br><br>*Use continuation sheet if necessary. Tick if continuation sheet used* ☐ *How many pages?* ☐ | | |
| **Date of onset/occurrence (DD-MMM-YYYY)** | __ __ - __ __ __ - __ __ __ __ | **Date resolved (DD-MMM-YYYY)**<br><br>**Or tick if ongoing** | __ __ - __ __ __ - __ __ __ __<br><br>☐ |

**Record details of investigations/relevant tests/laboratory data (including dates)**

| Investigation/test | Date | Result |
|---|---|---|
| | | |
| | | |
| | | |
| | | |
| | | |
| | | |
| | | |

**SECTION C: EVENT ASSESSMENT**

***When completing below please refer to definition guide at the end of this document***

**Action taken**

☐ None

☐ Medication given; specify ____________________

☐ Referred to hospital OPD/health facility; name of hospital/health facility ____________________

☐ Admitted as inpatient to hospital; name of hospital____________________

☐ Intervention (cash and/or cup) withdrawn

☐ Other; Specify____________________

**Outcome**

☐ Resolved

☐ Resolving/partly resolved

☐ Resolved with sequelae

☐ Not resolved

☐ Fatal

☐ Unknown

**Maximum severity**

☐ Mild

☐ Moderate

☐ Severe

☐ Life threatening

☐ Fatal

☐ Unable to assess

| Cups or Cash for Girls (CCg) Trial | | | | *Serious Adverse Event Form*<br>Sponsor: Liverpool School of Tropical Medicine, lstmgov@lstmed.ac.uk, pv@lstmed.ac.uk | |
|---|---|---|---|---|---|
| **Participant ID** | | **Date of report** | __ __ - __ __ __ - __ __ __ __ | **SAE no.** | |

| | | |
|---|---|---|
| **Seriousness** | ☐ Death<br><br>☐ Life threatening<br><br>☐ Hospitalisation<br><br>☐ Prolongation of existing hospitalisation<br><br>☐ Persistent or significant disability<br><br>☐ Other | ***If death:***<br><br>**Date of death (DD-MMM-YYYY)**<br><br>__ __ - __ __ __ - __ __ __ __ |
| | If other, specify | ***If hospitalisation:***<br><br>**Date admitted (DD-MMM-YYYY)**<br><br>__ __ - __ __ __ - __ __ __ __<br><br>**Date discharged (DD-MMM-YYYY)**<br><br>__ __ - __ __ __ - __ __ __ __ |

**SECTION D: INTERVENTION**

| | | | |
|---|---|---|---|
| **Cup** ☐ | **Cash** ☐ | **Cup & Cash** ☐ | **Usual practice (control)** ☐ |

*If the event is related to menstrual or vaginal health, complete the following questions:*

| | | | |
|---|---|---|---|
| **Was the girl having her monthly period at time of event?** | ☐ **Yes**<br>☐ **No** | **Did the girl have an item inserted in her vagina at time of event?** | ☐ **Yes**<br>☐ **No** |
| **If not at time of event, did she have an item inside her vagina on a recent day prior to the event (just removed)?** | ☐ **Yes**<br>☐ **No** | **Name of item which was inserted in vagina:** | ☐ **Rags** ☐ **Traditional medicine**<br>☐ **Grass** ☐ **Cup**<br>☐ **Newspaper** ☐ **Tissue paper**<br>☐ **Foam/bedding** ☐ **Cotton wool**<br>☐ **Sanitary pads** ☐ **Other; Specify** ________________ |

| Cups or Cash for Girls (CCg) Trial | | | ***Serious Adverse Event Form***<br>**Sponsor: Liverpool School of Tropical Medicine, lstmgov@lstmed.ac.uk, pv@lstmed.ac.uk** | | |
|---|---|---|---|---|---|
| **Participant ID** | | **Date of report** | __ __ - __ __ __ - __ __ __ __ | **SAE no.** | |

| **SECTION E: OTHER MEDICINE(S)/SUBSTANCE(S)** | |
|---|---|
| Were any other medicines taken up to two weeks prior to the onset of SAE? ☐ Yes ☐ No<br><br>*If yes, complete a concomitant drugs form and send with this SAE form* | |
| Were any traditional medicines taken up to two weeks prior to the onset of SAE? ☐ Yes ☐ No | |
| If yes, give details | |
| Were any other substances (e.g. alcohol, recreational drugs) taken in the two weeks prior to the onset of SAE? ☐ Yes ☐ No | |
| If yes, give details | |

| **SECTION F: CAUSALITY ASSESSMENT** | | |
|---|---|---|
| *Using the key below, insert number in the box to indicate causality for each intervention relevant to the patient. If the patient has not received the specified intervention, enter "7".*<br>*If you suspect the event is related to something **other** than the intervention(s), enter the causality in the 'other' box and provide further details on the continuation sheet.* | | |
| **Causality** | 1. Certain<br>2. Probable/likely<br>3. Possible<br>4. Unlikely<br>5. Unrelated<br>6. Unassessable<br>7. Not applicable (i.e. participant not receiving the intervention) | Cup ☐<br>Cash ☐<br>Other ☐ |

| **SECTION F: CAUSALITY ASSESSMENT *(continued)*** | | | |
|---|---|---|---|
| *If the answer to the above is 1, 2 or 3 for any intervention, complete the following assesments* ***FOR THE RELEVANT INTERVENTION ONLY*** | | | |
| **Action taken due to adverse event** | 1. Intervention/other withdrawn<br>2. Intervention/other withheld<br>3. None<br>4. Not applicable<br>5. Unknown | Cup | ☐ |
| | | Cash | ☐ |
| | | Other | ☐ |
| **De-challenge** | 1. Improved when intervention/other stopped<br>2. No improvement when intervention/other stopped<br>3. Not applicable (no de-challenge undertaken)<br>4. Unknown | Cup | ☐ |
| | | Cash | ☐ |
| | | Other | ☐ |
| **History with suspected intervention/other** | 1. Not used before<br>2. Used before, this event **did not** occur<br>3. Used before, this event **did** occur<br>4. Unknown | Cup | ☐ |
| | | Cash | ☐ |
| | | Other | ☐ |

| | | | |
|---|---|---|---|
| **SECTION G: REPORTER DETAILS** | | | |
| **Reporter Name** | | **Reporter Position** | |
| **Reporter Signature** | | **Date (DD-MMM-YYYY)** | __ __ - __ __ __ - __ __ __ __ |
| **SECTION H: PRINCIPAL INVESTIGATOR RECEIPT** | | | |
| **PI Name** | | | |
| **PI Signature** | | **Date (DD-MMM-YYYY)** | __ __ - __ __ __ - __ __ __ __ |

| Cups or Cash for Girls (CCg) Trial | | | ***Serious Adverse Event Form*** **Sponsor: Liverpool School of Tropical Medicine, lstmgov@lstmed.ac.uk, pv@lstmed.ac.uk** | | |
|---|---|---|---|---|---|
| **Participant ID** | | **Date of report** | __ __ - __ __ __ - __ __ __ __ | **SAE no.** | |

Appendix A. Body diagram – if appropriate, please indicate location of illness, infection, or injuries

| **APPENDIX A. BODY DIAGRAM** |
|---|
| *Please indicate with an arrow and words the location, and the type of illness, infection, or injuries on the diagram below (includes severe pain or bleeding)* |

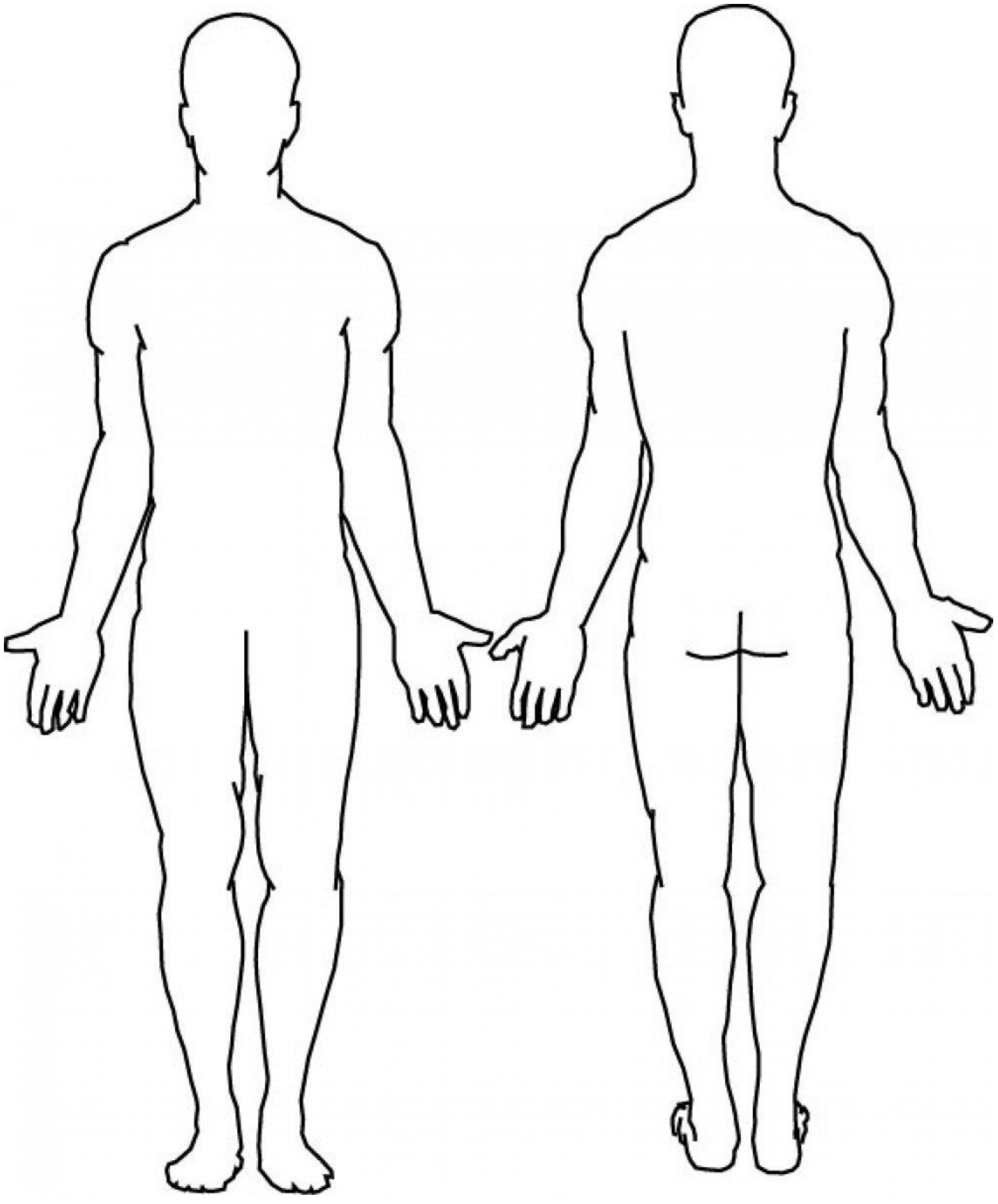

| Cups or Cash for Girls (CCg) Trial | | | | *Serious Adverse Event Form*<br>Sponsor: Liverpool School of Tropical Medicine, lstmgov@lstmed.ac.uk, pv@lstmed.ac.uk | | |
|---|---|---|---|---|---|---|
| Participant ID | | Date of report | | __ __ - __ __ __ - __ __ __ __ | SAE no. | |

| APPENDIX B. CONTINUATION SHEET |
|---|
| |
